# Quantifying the Survival of Multiple Salmonella enterica Serovars *In Vivo* via Massively Parallel Whole-Genome Sequencing To Predict Zoonotic Risk

**DOI:** 10.1128/AEM.02262-17

**Published:** 2018-01-31

**Authors:** Prerna Vohra, Marie Bugarel, Frances Turner, Guy H. Loneragan, Jayne C. Hope, John Hopkins, Mark P. Stevens

**Affiliations:** a The Roslin Institute and Royal (Dick) School of Veterinary Studies, University of Edinburgh, Easter Bush, Edinburgh, United Kingdom; b International Center for Food Industry Excellence, Department of Animal and Food Sciences, Texas Tech University, Lubbock, Texas, USA; c Edinburgh Genomics, University of Edinburgh, Easter Bush, Edinburgh, United Kingdom; University of Bayreuth

**Keywords:** Salmonella, cattle, whole-genome sequencing, zoonotic infections

## Abstract

Salmonella enterica is an animal and zoonotic pathogen of worldwide importance. Salmonella serovars that differ in their host and tissue tropisms exist. Cattle are an important reservoir of human nontyphoidal salmonellosis, and contaminated bovine peripheral lymph nodes enter the food chain via ground beef. The relative abilities of different serovars to survive within the bovine lymphatic system are poorly understood and constrain the development of control strategies. This problem was addressed by developing a massively parallel whole-genome sequencing method to study mixed-serovar infections *in vivo*. Salmonella serovars differ genetically by naturally occurring single nucleotide polymorphisms (SNPs) in certain genes. It was hypothesized that these SNPs could be used as markers to simultaneously identify serovars in mixed populations and quantify the abundance of each member in a population. The performance of the method was validated *in vitro* using simulated pools containing up to 11 serovars in various proportions. It was then applied to study serovar survival *in vivo* in cattle challenged orally with the same 11 serovars. All the serovars successfully colonized the bovine lymphatic system, including the peripheral lymph nodes, and thus pose similar risks of zoonosis. This method enables the fates of multiple genetically unmodified strains to be evaluated simultaneously in a single animal. It could be useful in reducing the number of animals required to study mixed-strain infections and in testing the cross-protective efficacy of vaccines and treatments. It also has the potential to be applied to diverse bacterial species which possess shared but polymorphic alleles.

**IMPORTANCE** While some Salmonella serovars are more frequently isolated from lymph nodes rather than the feces and environment of cattle, the relative abilities of serovars to survive within the lymphatic system of cattle remain ill defined. A sequencing-based method which used available information from sequenced Salmonella genomes to study the dynamics of mixed-serovar infections *in vivo* was developed. The main advantages of the method include the simultaneous identification and quantification of multiple strains without any genetic modification and minimal animal use. This approach could be used in vaccination trials or in epidemiological surveys where an understanding of the dynamics of closely related strains of a pathogen in mixed populations could inform the prediction of zoonotic risk and the development of intervention strategies.

## INTRODUCTION

Salmonella enterica is a bacterial pathogen of global importance for humans and animals. Depending on host and pathogen factors, infection with S. enterica can result in asymptomatic carriage, self-limiting diarrhea, or severe systemic typhoid-like disease. Cattle are a significant reservoir of S. enterica (1), harboring serovars which cause considerable morbidity and mortality in feedlots and also have the potential to cause human disease, primarily through contaminated food (2). Nontyphoidal Salmonella spp. are the leading cause of human foodborne illness and hospitalizations in the United States (3, 4) and were estimated to cause 78 million illnesses, 59,000 deaths, and a loss of 4 million disability-adjusted life years worldwide in 2010 (2). Approximately 7.3% of foodborne illnesses between 1998 and 2008 were attributed to beef products, and 7.2% of those illnesses were attributed to dairy products (5).

Salmonella is endemic in cattle in the United States. Studies have found that although the overall prevalence and the level of contamination in most beef products were low (6, 7), several serovars which cause disease in humans were isolated from them, including Salmonella enterica serovars Typhimurium, Montevideo, Dublin, and Newport (8–11). S. enterica serovars Newport and Typhimurium have also been directly implicated in outbreaks associated with ground beef (12–14). Cattle at slaughter have been reported to harbor more than one serovar, and even as many as 10 serovars, in their gut and lymph nodes (15). The threat to food safety posed by S. enterica-laden ileum and mesenteric lymph nodes (MLNs) is minimal, provided the gut is not perforated during slaughter, as these tissues are disposed of during the evisceration process. Peripheral lymph nodes (PLNs), however, are harder to remove on the scale of commercial beef production, as they are smaller and often located within adipose tissue. The prescapular (superficial cervical) and prefemoral (subiliac) lymph nodes are located in the chuck and flank, respectively, which are parts of the carcass that are routinely incorporated into ground beef. S. enterica serovars Typhimurium, Newport, Montevideo, Anatum, and Cerro, and even some multidrug-resistant isolates, have been detected in these lymph nodes in commercial cattle (16, 17), although the prevalence of Salmonella spp. can vary considerably between farms (18). One study found that while several serovars could be isolated from cattle at slaughter, there was a noticeable difference in the anatomical locations of different serovars (19). For instance, S. enterica serovar Meleagridis was more commonly isolated from PLNs, while S. enterica serovar Kentucky was more frequently isolated from hides and feces, suggesting that some serovars may be better adapted to enter and persist within the bovine lymphatic system, thus posing a greater threat to food safety.

Vaccines and interventions that effectively control gut colonization of cattle by S. enterica and the spread of serovars into the bovine lymphatic system are lacking, making this pathogen a major challenge for the beef industry. There are over 2,600 serovars of S. enterica, which makes it difficult to quantify the relative abilities of serovars to contaminate the bovine lymphatic system and predict which serovars to classify as high risk for bovine disease and zoonosis. Moreover, studying the virulence of serovars individually would be animal and resource intensive and, in the case of bovine salmonellosis, may not represent naturally occurring mixed infections.

**FIG 1:**
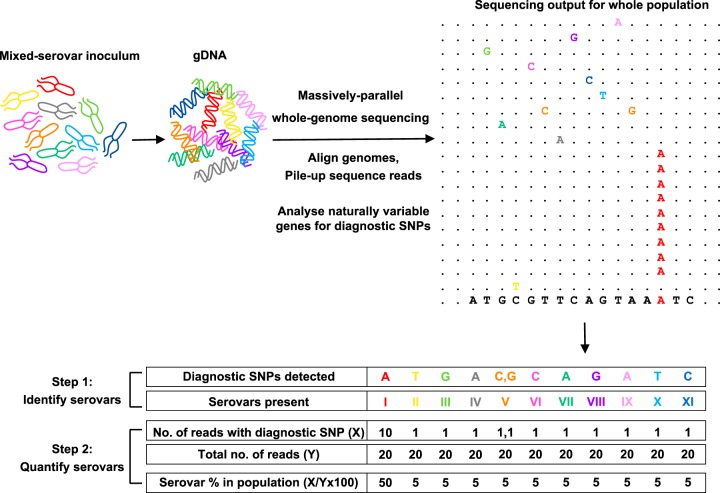
Approach to quantify bacterial strains in a mixed-strain population. Whole-genome sequencing followed by the analysis of known naturally variable genes for strain-specific SNPs can determine the composition of a mixed-strain population. S. enterica serovars can be distinguished by serovar-specific diagnostic SNPs in *rpoB* and *ileS*. When a mixed-serovar population is sequenced and aligned to a reference genome, these diagnostic SNPs can be used to identify the serovars in the population (step 1), and from the number of reads with diagnostic SNPs, the abundance of each serovar can be quantified (step 2). For example, here, the diagnostic SNP for serovar I (A) is detected 10 times out of a total 20 reads. Thus, it makes up 50% of the population. Diagnostic SNPs for the other serovars are detected only once. Thus, they each make up 5% of the population. When more than one diagnostic SNP is present, as seen for serovar V (C, G), the average abundance (5% at C and 5% at G) is used to estimate the overall abundance of that serovar in the population.

Serovars of S. enterica can be identified by various serological and molecular methods, such as serotyping (20), multiplex PCRs (21–23), and multilocus sequence typing (MLST) (24–26). They can be also distinguished at the genetic level by single nucleotide polymorphisms (SNPs) in conserved genes, such as *rpoB* (27), or hypervariable regions within them (28–30), and the discriminatory powers of other genes continue to be explored (31, 32). With whole-genome sequencing becoming routine for the surveillance of S. enterica (33–35), there is an ever-increasing database of sequenced strains. In this study, the inherent differences in the genomes of S. enterica serovars were exploited to develop a whole-genome sequencing method to study mixed-serovar infections without the need for traditional methods, such as colony subculture and serogrouping (Fig. 1). It was found that serovar-specific SNPs allowed the detection of multiple serovars present in a mixed population and their simultaneous quantification by the number of times each serovar-specific SNP was detected during sequencing of the entire population. The method was also applied to study the spread and survival of S. enterica serovars *in vivo*, within the gut and lymphatic system of cattle, in order to predict the zoonotic potential of different serovars.

## RESULTS

### Diagnostic SNPs can identify and quantify S. enterica serovars in mixed-serovar pools.

Sequence alignments of the *rpoB* and *ileS* genes (see Fig. S1 in the supplemental material) of the strains belonging to the 11 serovars studied here (Table 1) suggested that they had sufficient discriminatory power to distinguish between them. Between 3 and 23 serovar-specific nucleotide differences were identified in these two genes, referred to here as diagnostic SNPs (Table 2).

**TABLE 1:** S. enterica strains used in this study

Salmonella serovar	Serogroup	Strain	Origin
Dublin	D	SD3246	Bovine clinical isolate, UK, 1995
Typhimurium	B	ST4/74	Bovine clinical isolate, UK, 1966
Gallinarum	D	SG9	Fowl typhoid clinical isolate, UK, 1955
Montevideo	C1	09TTU806T	Bovine fecal isolate, USA, 2009
Newport	C2	09TTU1238R	Bovine fecal isolate, USA, 2009
Kentucky	C	09TTU1627T	Bovine fecal isolate, USA, 2009
Anatum	E	09TTU1944T	Bovine fecal isolate, USA, 2009
Agona	B	09TTU2919T	Bovine fecal isolate, USA, 2009
Meleagridis	E	12TTU1464B	Bovine lymph node isolate, USA, 2012
Cerro	K	11TTUT1136R	Bovine fecal isolate, USA, 2011
Reading	B	11TTUT0036T	Bovine fecal isolate, USA, 2011

**TABLE 2:** Serovar-specific diagnostic SNPs in *rpoB* and *ileS*

Salmonella serovar	Diagnostic SNPs in *rpoB*		Diagnostic SNPs in *ileS*		Total no. of diagnostic SNPs
	No.	Position/SNP	No.	Position/SNP	
Dublin	4	750/T, 780/G, 3117/A, 3864/A	3	1800/T, 1860/T, 2098/C	7
Typhimurium	6	456/C, 459/T, 588/A, 2634/A, 2944/A, 3330/C	1	1045/A	7
Gallinarum	4	140/G, 973/T, 3112/A, 3757/T	4	369/G, 426/T, 633/G, 2235/T	8
Montevideo	9	684/T, 1212/A, 1380/A, 1560/A, 2836/T, 3261/T, 3645/T, 3900/C, 3906/T	8	840/A, 918/T, 924/A, 1491/A, 1563/A, 1848/A, 1875/A, 2295/A	17
Newport	4	984/T, 1500/G, 1680/A, 3987/A	2	104/G, 2088/T	6
Kentucky	4	594/T, 1608/T, 1755/T, 3207/C	1	2116/A	5
Agona	1	3531/C	3	654/T, 2127/A, 2419/T	4
Anatum	4	1617/T, 2347/T, 2403/A, 2631/A	3	96/T, 1839/A, 2316/A	7
Meleagridis	3	1203/A, 1500/T, 3783/A	4	504/T, 537/T, 540/T, 875/A	7
Cerro	8	1938/T, 2634/T, 2661/A, 3294/A, 3336/C, 3339/T, 3414/T, 3564/T	15	273/A, 285/G, 609/T, 879/G, 972/T, 1014/T, 1017/T, 1041/A, 1050/T, 1059/C, 1254/C, 1506/C, 1683/C, 2181/A, 2376/A	23
Reading	3	882/C, 909/T, 972/A	0		3

**FIG 2:**
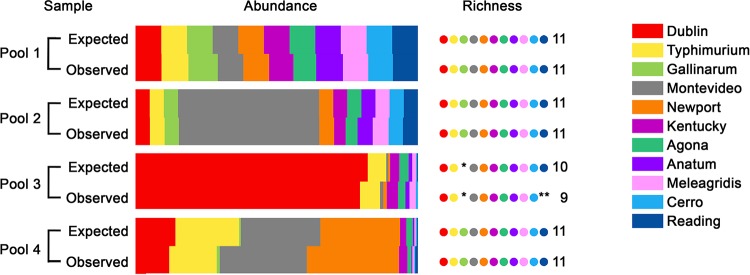
*In vitro* validation of serovar quantification using diagnostic SNPs. Four mixed-serovar pools containing up to 11 S. enterica serovars were prepared *in vitro* by mixing known quantities of separate cultures adjusted to the same optical density. The contributions of each serovar to the pool (abundance) are represented by color-coded bars. There was no significant difference between the population structures of any of the pools, as determined by viable counts (expected) and by sequencing and quantification of diagnostic SNPs (observed). The number of serovars present in the pool (richness) is indicated by dots. For pools 1, 2, and 4, all serovars were correctly identified as being present by sequencing. In pool 3, the asterisk indicates that *S*. Gallinarum, which was not added to the pool, was correctly identified as absent by sequencing. In the same pool, *S*. Reading was present at the limit of detection, which was a single read for each diagnostic SNP, and the double asterisks indicate that it was not detected by sequencing. However, it was detected accurately in pool 4 at the same limit of detection of a single read.

To determine if the diagnostic SNPs could be used to accurately identify and quantify serovars in mixed populations, the method was applied to 4 simulated pools of known composition. Following bioinformatics analysis (Fig. S2), the compositions of the pools, as determined by viable counts and sequencing, were compared (Fig. 2). The expected read numbers were calculated using the percentages of the serovars in the pools and the actual read depth across the diagnostic SNP positions during each run, as read depth varied between runs (Fig. S3 and S4). These observed read numbers were obtained from the sequencing output (Table S1). No statistically significant differences in expected and observed read numbers were obtained for any of the pools (pool 1, *c*^2^_10_ = 1.842, *P* = 0.9974; pool 2, *c*^2^_10_ = 1.664, *P* = 0. 9983; pool 3, *c*^2^_8_ = 7.985, *P* = 0.4349; pool 4, *c*^2^_10_ = 11.08, *P* = 0.3512), which validated the performance of the method. This also showed that the enrichment of pools on MacConkey agar prior to sequencing did not introduce any growth rate-related bias into the screen, making this approach suitable for *in vivo* studies.

Additional parameters were tested using these pools. To test the specificity of the method, S. enterica serovar Gallinarum was not included in pool 3; consequently, its corresponding diagnostic SNPs were not detected by sequencing. To test the sensitivity of the method, S. enterica serovars Cerro and Reading were added to pools 3 and 4, such that they made up only 0.33% and 0.34% of the pools, respectively, which would result in obtaining a single diagnostic SNP-containing read for each serovar at the assumed maximum sequencing depth. In pools 3 and 4, 1.26 ± 0.30 and 0.87 ± 0.17 reads, respectively, were obtained for *S*. Cerro across its diagnostic SNPs. For *S*. Reading, while 2.3 ± 0.27 reads were obtained in pool 4, none were detected in pool 3, showing that the sensitivity of the method depends on the depth of sequencing used.

### Diagnostic SNPs can resolve S. enterica population structures *in vivo*.

**FIG 3:**
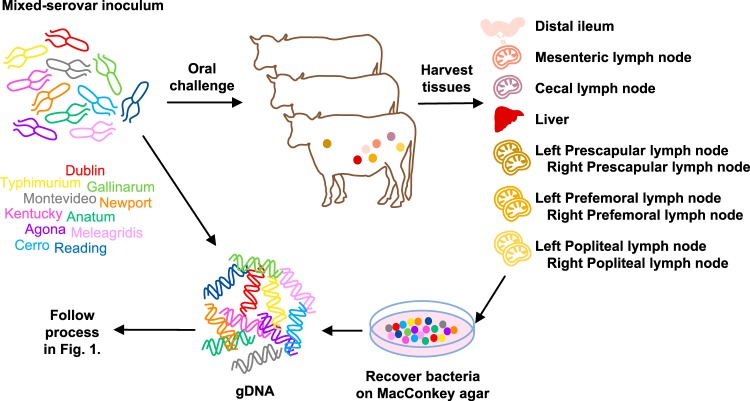
*In vivo* experimental design. To study the survival of S. enterica serovars *in vivo*, calves were orally challenged with an inoculum containing 11 serovars in equal proportions. At 48 h postinfection, bacteria were recovered from the distal ileum, MLNs, CLNs, liver, and the left and right prescapular, prefemoral, and popliteal PLNs by enrichment on MacConkey agar. Bacterial lawns were scraped from agar plates, and gDNA was extracted using a standard commercial kit. Whole-genome sequencing and bioinformatics analysis were performed as described in Fig. 1 to determine the compositions of the Salmonella populations recovered from each tissue.

The method was then applied to study mixed-serovar Salmonella infection *in vivo*. Calves challenged orally with an inoculum containing all 11 serovars (Fig. 3) showed clinical signs of salmonellosis, indicated by pyrexia, diarrhea, and decreased food intake by 36 h postinfection, as expected (36, 37), and were humanely killed at 48 h postinfection. Bacterial recoveries from the tissues were consistent across the three calves (Fig. S5). The distal ileum, MLNs, and cecal lymph nodes (CLNs) contained approximately 10^6^ CFU of Salmonella per gram. In contrast, the PLNs and livers yielded only approximately 300 CFU per gram. Irrespective of the bacterial load, lawns of Salmonella were recovered on MacConkey agar from all the tissues, which were processed in the same manner as the *in vitro* pools.

**FIG 4:**
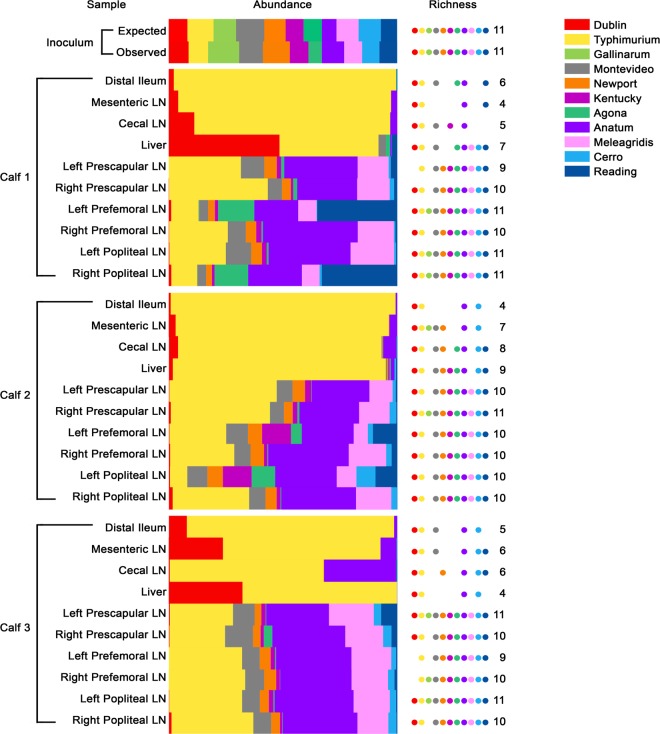
Salmonella populations recovered from the tissues of infected calves. Calves were orally challenged with an inoculum containing 11 S. enterica serovars in approximately equal proportions. The contributions of each serovar to the population in each tissue (abundance) are represented by color-coded bars, and the number of serovars present in each pool (richness) is indicated by dots. The composition of the inoculum as determined by viable counts (expected) was not significantly different from that determined by sequencing and quantification of diagnostic SNPs (observed). In the distal ileum, MLNs, and CLNs of all 3 calves, *S*. Typhimurium was the predominant serovar, and these tissues had less richness than the PLNs. Salmonella serovars Typhimurium and Dublin were most abundant in the livers of all the animals. The PLNs (prescapular, prefemoral, and popliteal lymph nodes) had richness scores of at least 10, indicating that all the serovars could reach and survive within these tissues by 48 h postinfection. Salmonella serovars Typhimurium and Anatum were most abundant across all the PLNs.

To confirm that the calves had been challenged with the desired inoculum, the observed and expected reads for the 11 serovars in the inoculum were compared as described before, and no statistically significant difference was obtained (*c*^2^_10_ = 15.16, *P* = 0.1262). Diverse Salmonella populations were recovered from the tissues of the three infected calves, and their structures were successfully resolved using this method (Fig. 4).

### Salmonella serovars exhibit similar zoonotic potentials.

The Salmonella populations recovered from the distal ileum of all the calves had richness scores between 4 and 6, indicating that most serovars were not detected at 48 h postinfection. The *S*. Typhimurium strain ST4/74 was predominant (95.22% ± 2.48%), followed by *S*. Dublin, albeit at a much lower percentage (3.53% ± 1.72%); other serovars were present at ≤1.3%. There was a striking predominance of *S*. Typhimurium in MLNs and CLNs as well (82.69% ± 4.30%), followed by the strains of *S*. Dublin and *S*. Anatum (7.74% ± 3.25% and 8.85% ± 4.17%, respectively); other serovars were present at ≤0.32%. The richness scores of the populations in the livers of calves 1, 2, and 3 were 7, 9, and 4, respectively. The liver is often used as an indicator of systemic Salmonella infection. Here, too, *S*. Typhimurium was predominant (68.11% ± 11.93%), consistent with the known biology of this strain in calves of the age and breed used in this study (37). In two calves, *S*. Dublin SD3246 was appreciably enriched in the liver compared to the gut, consistent with the known systemic virulence of this strain, while *S*. Gallinarum SG9 was absent from the liver in all calves, as expected (37, 38).

The Salmonella populations recovered from the PLNs, however, were very different from those recovered from the other tissues. Across the PLNs of all three calves, although *S*. Typhimurium (28.75% ± 2.50%) and *S*. Anatum (29.74% ± 1.26%) were consistently predominant, their abundances were less than those in the ileum or lymph nodes. Other serovars contributed more to the PLN populations, such as *S*. Meleagridis (13.70% ± 0.95%), *S*. Montevideo (7.80% ± 0.50%), *S*. Reading (5.95% ± 2.40%), *S*. Newport (4.50% ± 0.30%), *S*. Agona (3.03% ± 1.12%), *S*. Kentucky (2.80% ± 0.72%), *S*. Cerro (2.40% ± 0.41%), and *S*. Dublin (0.44% ± 0.11%). Interestingly, *S*. Gallinarum SG9 was isolated from some PLNs but at extremely low percentages (0.04% ± 0.02%), while *S*. Dublin SD3246, despite being known to cause typhoid-like disease in cattle, was not always detected. A most significant finding was that the richness score for all the PLNs was at least 10, indicating that all the serovars could colonize and survive within the PLNs by 48 h after oral inoculation. Interestingly, the Salmonella populations in PLNs from one side of a calf were not necessarily mirrored in those from the other side.

## DISCUSSION

In this study, the sequence diversity among S. enterica serovars identified *in silico* was used to study the dynamics of mixed-serovar infections *in vivo*. By performing whole-genome sequencing and using serovar-unique diagnostic SNPs in just two genes as markers, unmodified strains of at least 11 serovars of S. enterica were accurately distinguished within mixed populations, and their survival within the bovine lymphatic system was quantified to predict their zoonotic potential.

Technically, there are many advantages to using this method. As entire genomes are sequenced, there is no limit to the number of regions that can be used to distinguish serovars or strains from each other, which is an improvement over PCR amplification of specific genes. Indeed, where multilocus sequence typing (MLST) is effective at distinguishing clonal groups, the analysis could be conducted on the housekeeping genes that are routinely used. The lack of PCR also reduces the time and cost of the method, although it is feasible that amplification of polymorphic regions followed by deep sequencing may enable more sensitive detection of less-abundant population members. The use of multiple diagnostic SNPs for each strain, as described here, serves as an internal control for variations in read depth and adds to the robustness of the method.

The recovery of bacterial lawns from infected tissues ensures that the population diversity within samples is maintained and allows for amplification of minority members that may be difficult to detect directly in tissues, as long as growth rates of the strains do not vary considerably on the recovery medium and this is tested prior to any *in vivo* studies, as done here. This is particularly relevant for tissues such as the PLNs in this study, which typically have low levels of bacterial contamination. Conversely, it could be argued that minority population members might be missed in larger samples. While this is possible, members comprising as little as 0.34% of a pool in this study, which corresponded to a single sequencing read at the depth of sequencing used, were accurately detected. The prospects to detect rare and minor population members could be improved by increasing the depth of sequencing but at added cost.

A major advantage of this approach is the ability to study bacterial strains without carrying out any genetic manipulation. Previous studies on the spatiotemporal distribution of S. enterica infections *in vivo* have used chromosomal tags detected by quantitative PCR to track the strains being studied (39, 40), which requires a conserved and inert chromosomal region and genetically tractable strains. The conserved and inert chromosomal region is not always available across serovars; for example, the region between the pseudogenes *malX* and *malY* targeted in *S*. Typhimurium (40) is intact in only some serovars, while bacterial field isolates are often more difficult to manipulate, and a risk of spontaneous mutation on repeated laboratory passage exists. Further, this approach omits the need for serotyping or sequencing large numbers of colonies separately. It also allows strains belonging to the same serogroup, which would be indistinguishable by conventional serotyping, to be studied simultaneously, as demonstrated here.

Another advantage of this method is the significant reduction in the number of animals required to study mixed infections *in vivo*, in keeping with the principles of the 3Rs (replacement, reduction, and refinement), which guide the ethical use of animals in research (41, 42). As the fate of multiple serovars can be followed within a single animal simultaneously, the need to study serovars individually is eliminated. Here, just 3 calves were used to assign phenotypes to 11 strains *in vivo*, whereas at least 33 animals would have been required to test them individually (assuming the use of 3 calves per strain to account for interanimal variance based on past studies).

Some key observations regarding serovar behavior *in vivo* were made during this study. Notably, previously described patterns of *in vivo* spread of three strains of well-defined virulence, which were included as internal controls, were observed in this study. Single-serovar-infection studies using age-matched calves of the same breed have shown that the cattle-adapted *S*. Dublin SD3246 causes systemic typhoid-like disease and persists in the liver; the avian pathogen *S*. Gallinarum SG9 can effectively invade the bovine gut but is avirulent in calves; and the generalist *S*. Typhimurium ST4/74, which causes acute enteritis in calves, can replicate rapidly and be more invasive than SD3246 in the bovine gut (37, 38, 43). The enhanced replication of ST4/74 compared to host-adapted S. enterica serovar Choleraesuis has been observed in porcine intestines as well (44). Similar observations in calves infected with a mixed inoculum here lend confidence to the observations on the *in vivo* spread of the other 8 serovars. It also suggests that a virulent strain does not necessarily rescue the phenotype of an avirulent strain via effects on the host or interbacterial interactions.

A significant finding was that all the serovars were detected in the PLNs and thus have the potential to enter the food chain through ground beef products and affect human health. Therefore, the development of any intervention against Salmonella in cattle is likely to require a panserovar approach. This observation was consistent across animals, suggesting that host factors or stochastic processes did not affect the distribution of serovars within the PLNs. It is possible that all the Salmonella serovars studied here reached the PLNs by passive carriage. Indeed, previous studies have shown the rapid translocation of Salmonella from the bovine gut into the lymphatics in a cell-free state (38) and the lack of population bottlenecks restricting this transit, at least when large inocula are used (45, 46). However, the fact that all the serovars survived within the lymphatics and were recovered from the PLNs demonstrated their potential to pose a risk to food safety.

Finally, the Salmonella populations in the gut and MLNs were markedly different from those in the PLNs. Fewer serovars were detected in these tissues, perhaps as a result of the rapid outgrowth of *S*. Typhimurium, the relative susceptibility of other serovars to host defenses, or a failure to detect serovars present at very low percentages. In any case, this suggests that despite the ease of access of the gut and MLNs at postmortem examination, they cannot be used as representative tissues to understand the behavior and interactions of Salmonella serovars in the entire lymphatic system. Also, the population differences observed within PLN pairs suggest that sampling just one branch of the lymphatic system may misrepresent the true nature of salmonellosis within an animal. It is important for future surveys of Salmonella prevalence in livestock to account for this variation to obtain a representative picture of serovar survival *in vivo*.

The limitations of this study include the use of a single strain from each S. enterica serovar and a single time point for tissue sampling. Thus, the behavior of other strains of these serovars *in vivo* or their temporal distribution in different tissues cannot be commented on conclusively. However, the approach could be adapted to study multiple strains of the same serovar, provided that strain-specific SNPs are found. Also, animals challenged with the same inoculum could be sacrificed at a series of time points to study possible changes in Salmonella populations in tissues over time, due to either sequential colonization of lymph nodes or greater replication of some strains. However, a previous study in which calves were challenged simultaneously with *S*. Dublin, *S*. Typhimurium, and *S*. Choleraesuis showed that following rapid early colonization of the gut and MLNs by all 3 serovars, there was no considerable change in their individual abundances in the liver and contents of the small intestine over the 7 days of sampling (36).

This method has the potential to be used not only to study mixed infections but also to evaluate vaccines or treatments for their ability to cross-protect against prevalent serovars of Salmonella or other pathogens for which it could be adapted. Previous such work has relied on biochemical tests and serogrouping to identify the Salmonella species recovered from vaccinated animals (47–49) or has been restricted to testing one strain at a time (50–52). These limitations could be overcome by using this method. Another possible application is the monitoring of Salmonella or other pathogens from the farm during processing and in food products or the environment. Whole-genome sequencing has already been applied to Salmonella surveillance, but it has involved sequencing multiple isolates singly to aid the prediction of zoonotic risk (33, 53).

The advent of inexpensive whole-genome sequencing has led to an explosion in the volume of sequence data for pathogens. At the time of writing, almost 100,000 sequences are available for S. enterica in EnteroBase (http://enterobase.warwick.ac.uk) alone. To understand the consequences of the genetic variation observed among such strains, methods are required to evaluate virulence in relevant animal models with minimal animal use and harm. Here, the potential to use whole-genome sequencing and the investigation of polymorphic alleles to study mixed-strain infections *in vivo* has been demonstrated. While this was done using serovars of S. enterica to study salmonellosis in cattle, it could be applied to any bacterial pathogen for which there is a database of known genome sequences in which multiple, unique, and reliable strain-specific polymorphisms are identified.

## MATERIALS AND METHODS

### Bacterial strains and culture conditions.

Salmonella enterica strains were routinely cultured at 37°C in Luria-Bertani (LB) broth and on MacConkey agar without antibiotic selection. Strains of 11 serovars were used in this study (Table 1). These included 3 isolates of well-defined virulence in calves—*S*. Dublin SD3246 (GenBank accession no. CM001151), *S*. Typhimurium ST4/74 (accession no. CP002487), and *S*. Gallinarum SG9 (accession no. CM001153) (37, 55)—and single isolates of Salmonella serovars Montevideo, Newport, Kentucky, Agona, Anatum, Meleagridis, Cerro, and Reading, which were isolated from cattle in the United States.

### Preparation of mixed-serovar pools.

Bacterial cultures were prepared by inoculating several colonies in LB broth and incubating statically at 37°C for 16 h. Cultures of each serovar were standardized to an optical density at 600 nm (OD_600_) to give 10^8^ CFU · ml^−1^, which was confirmed by retrospective plating of 10-fold serial dilutions and determination of viable counts. Four mixed-serovar pools of known composition were prepared *in vitro* (Table S1). The volumes of cultures in the pools were adjusted such that, at the assumed maximum depth of sequencing (360×), a range of serovar abundances within populations would be tested. Pool 1 was prepared by mixing equal volumes of all 11 cultures to mimic a mixed-serovar inoculum, as would be used for *in vivo* testing; thus, it contained each serovar at 9.09%. Pools 2, 3, and 4 were prepared to mimic potential output pools obtained from infected animal tissues and contained serovars at percentages ranging from 0.33 to 82.28%. Pools 2, 3, and 4 were additionally spread plated onto 10 MacConkey agar plates each (500 μl · plate^−1^), as would occur following the recovery of Salmonella from infected tissues, and incubated overnight at 37°C. The resulting bacterial lawns were collected by washing with phosphate-buffered saline (PBS), and the pellets were stored at −20°C for DNA extraction.

### Identification of diagnostic SNPs.

The gene sequences of *rpoB* and *ileS* of the 11 strains used in this study were obtained from published data and sequencing performed by the coauthors at Texas Tech University (accession numbers MG457308 to MG457323). Sequences were aligned using Multalin (56), and nucleotide differences were recorded manually (Table 2). These serovar-specific diagnostic SNPs were used to identify and quantify serovars in mixed pools. A cladogram of concatenated *rpoB* and *ileS* sequences was generated using MUSCLE (57) (Fig. S1).

### Oral infection of calves with a mixed-serovar inoculum.

Animal experiments were conducted according to the requirements of the Animals (Scientific Procedures) Act 1986 (license PPL 60/4420) with the approval of the local ethical review committee. Twenty-eight-day-old Friesian bull calves were housed in a secure animal unit and fed a diet of fresh milk. Calves were confirmed to be culture negative for Salmonella before inoculation by enrichment of fecal samples in Rappaport-Vassiliadis broth at 37°C for 18 h, followed by plating on MacConkey agar at 37°C for 24 h. The inoculum for the oral challenge of calves was prepared such that 20 ml contained 10^10^ CFU of each serovar, which was confirmed by retrospective plating of 10-fold serial dilutions and determination of viable counts. Aliquots of the inoculum were stored at −20°C for DNA extraction. Twenty milliliters of the inoculum was mixed with 20 ml of antacid [5% Mg(SiO_3_)_3_, 5% NaHCO_3_, and 5% MgO in sterile distilled water] to promote colonization and administered orally to 3 calves by syringe before the morning feed. Calves were fed as normal following challenge and were monitored every 12 h, as previously described (58). Postmortem examinations were performed at 48 h postinfection.

### Sample collection.

A section of distal ileal mucosa, MLNs draining the distal ileal loop, CLNs, liver, and PLNs were collected. The PLNs included the left and right prescapular, prefemoral, and popliteal lymph nodes. Instruments were changed for each site sampled, and tissues from the gut were removed last to avoid cross-contamination. Samples were collected in an isotonic medium (0.75% choline chloride, 0.27% KCl, 1.8% glucose, 0.5% choline bicarbonate, 1% 10× Eagle minimum essential medium with Earle's salts, 1% fetal calf serum, 20 mM l-glutamine, and 0.3% NaHCO_3_ in distilled water) and transported to the laboratory on ice.

### Bacteriological analysis of tissues.

Lymph nodes were trimmed of excess fat and fascia, and the section of distal ileum was washed gently in PBS to remove nonadherent bacteria. One gram of MLNs and CLNs and a 1-g full-thickness biopsy specimen of distal ileum were homogenized in 8 ml of PBS in gentleMACS M tubes using the appropriate setting on the gentleMACS dissociator (Miltenyi Biotec). Host cells were lysed by the addition of 1 ml of 10% Triton X-100, and tissue debris was removed by filtering through 40-μm-pore-size filters. Tenfold serial dilutions were plated on MacConkey agar to determine viable counts. The remaining homogenate was spread onto 10 MacConkey agar plates (500 μl · plate^−1^) and incubated overnight at 37°C. The resulting bacterial lawns were collected by washing with PBS, and the pellets were stored at −20°C for DNA extraction. For the PLNs and livers, entire lymph nodes and up to 3 g of tissue, respectively, were homogenized. Infrequently, the bacterial lawns contained lactose-fermenting bacteria, and contamination was minimized either by physically removing the colonies or by generating lawns from a lower dilution of tissue.

### DNA extraction and sequencing.

Genomic DNA (gDNA) was extracted from the bacterial pellets stored at −20°C using the NucleoSpin tissue kit (Macherey-Nagel), according to the manufacturer's instructions. The quality and quantity of DNA were assessed initially by NanoDrop 3300 (Thermo Scientific), and samples with an *A*_260/280_ of ≤1.8 were considered suitable for library preparation. The quality and quantity of DNA were confirmed using the DNA ScreenTape (Agilent Technologies) and the Qubit double-stranded DNA (dsDNA) BR assay kit (Life Technologies), respectively. One microgram of gDNA with a DNA integrity number (DIN) of ≤6 was used for library preparation using the TruSeq PCR-free library preparation kit (Illumina) according to the manufacturer's protocol. The gDNA was sheared to a median size of 550 bp using an E220 Focused-ultrasonicator (Covaris), according to the manufacturer's recommended settings, and used as input to the Illumina library preparation kit. Briefly, the gDNA was end repaired and size selected using magnetic beads. The blunt-ended gDNA was A-tailed by the addition of an adenosine group to the 3′ ends. Barcoded sequencing adapters were ligated to the fragments, and unligated material was removed by 2 rounds of DNA cleanup using magnetic beads to produce the final library. The quality of each library was assessed using electrophoresis on the D1000 ScreenTape (Agilent), and the quantity of each library was assessed using quantitative PCR (qPCR) with the Kapa library quantification kit (Kapa Biosciences). The gDNA libraries were normalized to a concentration of 7 nM, pooled in equimolar concentrations, and loaded onto a MiSeq or HiSeq system (Illumina) at a concentration of 9 pM for sequencing. Paired-end sequencing was carried out using a 300-cycle kit for 150 cycles. Pools 1 and 2 were sequenced in a single lane of the MiSeq. Pools 3 and 4 were sequenced similarly in an independent run. Samples from the calf infection experiment were multiplexed, and 15 to 16 samples were sequenced in a single lane of the HiSeq.

### Bioinformatics analysis.

The bioinformatics workflow applied to the sequencing data generated is described in Fig. S2. Briefly, sequencing reads were aligned to the *S*. Dublin SD3246 genome (accession no. CM001151) using Burrows-Wheeler Aligner (BWA) sampe version 0.7.13 (59). Duplicate reads were marked using MarkDuplicates in Picard Tools version 1.115 (Broad Institute) and filtered using the command “samtools view -F 1024” (60). Local realignment around indels was performed using the IndelRealigner tool in GATK version 3.4.0 (61). One bam file was generated for each sample. The data were converted into pileup format using the SAMtools (62) version 1.2 “mpileup” command. PoPoolation2 (63) was used to generate a “sync” file from the pileup format using mpileup2sync.jar with the parameter -q20, which ensured that bases with a base call quality of <20 were discarded. The BLASTN program Megablast (64) version 2.2.28 was used to identify the genome coordinates of *rpoB* and *ileS* and the diagnostic SNPs within them for the 11 serovars. The total number of reads (read depth) at each diagnostic SNP position was recorded (Fig. S3 and S4). From the number of reads observed with diagnostic SNPs, allele frequencies for each serovar were calculated. Allele frequency was calculated as the number of reads with diagnostic SNP divided by the total number of reads. For samples recovered from infected tissues, occasionally, a small number of SNPs showed an allele frequency greater than those of other diagnostic SNPs for the same serovar within the same population. SNPs with an allele frequency of >1.5 times the mean frequency of other diagnostic SNPs and >4% higher in at least 2 populations for the same serovar were discarded. As there was more than one diagnostic SNP for each serovar, quantification was still possible after removing these SNPs. For each serovar, the average allele frequency across multiple SNPs was calculated. The serovar percentage in a population was calculated as the average allele frequency × 100. Data are presented as the mean ± standard error of the mean (SEM).

### Statistical analysis.

The expected percentage of each serovar in a population, as determined by viable counts, was transformed into the number of diagnostic SNP-containing reads expected for that serovar using the actual depth of sequencing across all the diagnostic SNP positions for that serovar in each sequencing run (Table S1). The mean of the observed number of diagnostic SNP-containing reads for each serovar was determined from the bioinformatics analysis. Expected and observed read numbers were rounded to the closest integer and compared using the chi-square (*c*^2^) test, excluding any values that were 0. Statistical tests were performed in GraphPad Prism version 7.00 (GraphPad Software). *P* values of ≤0.05 were considered to be statistically significant.

### Accession number(s).

The sequences of *rpoB* and *ileS* of the strains belonging to Salmonella serovars Montevideo, Newport, Kentucky, Agona, Anatum, Meleagridis, Cerro, and Reading used in this study have been deposited to GenBank under accession numbers MG457308, MG457309, MG457310, MG457311, MG457312, MG457313, MG457314, and MG457315 (*rpoB*) and MG457316, MG457317, MG457318, MG457319, MG457320, MG457321, MG457322, and MG457323 (*ileS*).

## Supplementary Material

Supplemental material
