# Supplementary material for: Quantifying the Survival of Multiple Salmonella enterica Serovars In Vivo via Massively Parallel Whole-Genome Sequencing To Predict Zoonotic Risk
Source: Appl Environ Microbiol. 2018 Jan 31;84(4):e02262-17. doi: 10.1128/AEM.02262-17 (PMC5795071; doi:10.1128/AEM.02262-17)
Supplement: Supplemental material [file supp_84_4_e02262-17__index.html]

Supplemental material 

# Quantifying the Survival of Multiple Salmonella enterica Serovars *In Vivo* via Massively Parallel Whole-Genome Sequencing To Predict Zoonotic Risk

## Supplemental material

- Supplemental file 1 -

  Expected and observed compositions of mixed-serovar pools (Table S1); cladogram of concatanated *rpoB*-*ileS* sequences (Fig. S1); bioinformatics analysis workflow for this study (Fig. S2); read depths (Fig. S3 and S4); bacterial loads in tissues of infected calves (Fig. S5).

  PDF, 368K
