# Supplementary material for: Quantifying the Survival of Multiple Salmonella enterica Serovars In Vivo via Massively Parallel Whole-Genome Sequencing To Predict Zoonotic Risk
Source: Appl Environ Microbiol. 2018 Jan 31;84(4):e02262-17. doi: 10.1128/AEM.02262-17 (PMC5795071; doi:10.1128/AEM.02262-17)
Supplement: Supplemental material [file AEM.02262-17_zam004188303s1.pdf]

Supplementary Tables

TABLE S1 Expected and observed compositions of simulated *in vitro* mixed-serovar pools

	Serovar	Expected %	Expected no. of reads with diagnostic SNPs at		Observed no. of reads with diagnostic SNPs[c] (mean ± SEM)	Observed %
			360X[a] (For pool prepartion)	Actual read depth[b] (For analysis)		
Pool 1	Dublin	9.09	33	44	41.43 ± 1.87	9.20
Pool 1	Typhimurium	9.09	33	46	44.57 ± 3.49	9.90
Pool 1	Gallinarum	9.09	33	42	46.00 ± 3.77	10.21
Pool 1	Montevideo	9.09	33	42	40.88 ± 1.97	9.07
Pool 1	Newport	9.09	33	42	39.83 ± 1.85	8.85
Pool 1	Kentucky	9.09	33	41	37.80 ± 3.34	8.41
Pool 1	Anatum	9.09	33	42	35.75 ± 3.47	7.93
Pool 1	Agona	9.09	33	42	42.71 ± 1.40	9.48
Pool 1	Meleagridis	9.09	33	43	39.43 ± 3.12	8.75
Pool 1	Cerro	9.09	33	42	39.31 ± 1.58	8.73
Pool 1	Reading	9.09	33	45	42.67 ± 2.60	9.47
Pool 2	Dublin	5.00	18	22	19.43 ± 1.79	4.77
Pool 2	Typhimurium	5.00	18	23	23.00 ± 1.12	5.64
Pool 2	Gallinarum	5.00	18	21	20.63 ± 2.13	5.07
Pool 2	Montevideo	50.00	180	204	199.24 ± 6.56	48.93
Pool 2	Newport	5.00	18	21	21.17 ± 2.42	5.20
Pool 2	Kentucky	5.00	18	21	16.80 ± 2.88	4.13
Pool 2	Anatum	5.00	18	22	17.50 ± 2.05	4.30
Pool 2	Agona	5.00	18	21	21.57 ± 1.84	5.30
Pool 2	Meleagridis	5.00	18	21	22.57 ± 2.77	5.53
Pool 2	Cerro	5.00	18	21	20.30 ± 4.99	4.99
Pool 2	Reading	5.00	18	24	25.00 ± 1.41	6.14
Pool 3	Dublin	82.24	296	236	229.00 ± 6.02	80.72
Pool 3	Typhimurium	6.58	24	18	19.14 ± 1.28	6.74
Pool 3	Gallinarum	0.00	0	0	0.00	0.00
Pool 3	Montevideo	0.66	2	2	3.06 ± 0.43	1.10
Pool 3	Newport	0.66	2	2	3.40 ± 0.30	1.18
Pool 3	Kentucky	3.29	12	9	10.40 ± 1.15	3.66
Pool 3	Anatum	3.29	12	10	7.50 ± 1.60	2.63
Pool 3	Agona	1.32	5	4	3.86 ± 0.84	1.36
Pool 3	Meleagridis	1.32	5	4	6.14 ± 0.91	2.16
Pool 3	Cerro	0.33	1	1	1.26 ± 0.30	0.45
Pool 3	Reading	0.33	1	1	0.00	0.00
Pool 4	Dublin	14.11	51	40	32.86 ± 2.35	11.93
Pool 4	Typhimurium	22.57	81	65	47.43 ± 2.04	17.22
Pool 4	Gallinarum	0.56	2	2	1.13 ± 0.41	0.41
Pool 4	Montevideo	28.22	102	80	86.82 ± 2.91	31.53
Pool 4	Newport	28.22	102	77	88.67 ± 4.39	32.20
Pool 4	Kentucky	2.26	8	7	8.40 ± 2.05	3.05
Pool 4	Anatum	2.26	8	7	4.00 ± 1.06	1.45
Pool 4	Agona	0.56	2	2	1 ± 0.50	0.36
Pool 4	Meleagridis	0.56	2	2	1.86 ± 0.62	0.68
Pool 4	Cerro	0.34	1	1	0.87 ± 0.17	0.32
Pool 4	Reading	0.34	1	1	2.33 ± 0.27	0.85

[a] A maximum sequencing depth of 360X was assumed in order to prepare the pools, such that serovars would be present at 0.33%-82.24%, corresponding to 1-296 reads with diagnostic SNPs, respectively.

[b] Following sequencing, it was observed that the actual read depth differed from the assumed read depth. The expected number of reads with diagnostic SNPs for each serovar were re-calculated using the average read depth across all the diagnostic SNP positions for each serovar, which were then compared to the observed read numbers for statistical analysis.

[c] The observed number of reads with diagnostic SNPs was calculated across several diagnostic SNP positions for each serovar and is thus, represented as a mean ± SEM.

Supplementary Figures

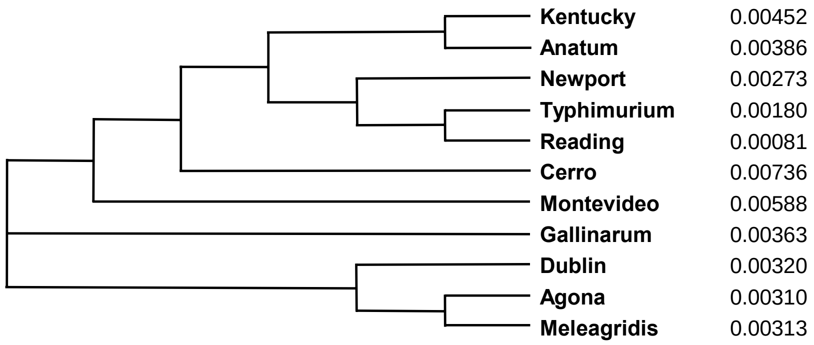

FIG S1 Cladogram of concatanated *rpoB-ileS* sequences. The gene sequences of *rpoB* and *ileS* of the 11 strains used in this study have sufficient SNPs in them to be able to distinguish *S. enterica* serovars from each other. The values shown beside the serovar names indicate the proportions of diagnostic SNPs identified in the sequences of *rpoB* and *ileS* for that serovar.

Step 1: Identify serovars

1. Align reads to *S*. Dublin SD3246 genome (CM001151.1)
[Burrows Wheeler Aligner sampe version 0.7.13]

2. Mark duplicate reads
[Picard tools version 1.115, tool "MarkDuplicates"]

3. Filter duplicate reads
[Picard tools version 1.115, command "samtools view -F 1024"]

4. Perform local realignment
[GATK version 3.4.0, tool "IndelRealigner2]

5. Convert data into pileup format
[SAMtools version 1.2, command "mpileup"]

6. Generate sync file and discard bases with a base call quality of <20
[PoPoolation2, parameter "-q 20"]

7. Convert positions of diagnostic SNPs from gene positions to genome coordinates. Identify serovars present from user-provided coordinates of diagnostic SNPs (Table 1)
[BLASTN megablast version 2.2.28]

↓

Step 2: Quantify serovars

8. Calculate allele frequencies at each diagnostic SNP (Table 1) for all serovars
[No. of reads containing diagnostic SNP/Total no. of reads at that position]

9. Remove erroneous diagnostic SNPs from analysis
[SNPs with allele frequency >1.5 in at least 2 populations compared to mean frequency]

10. Calculate % of each serovar present (mean±SEM) in population
[Average allele frequency across all diagnostic SNPs x 100]

FIG S2 Bioinformatics analysis workflow for this study. Sequence reads were aligned to the genome of *S*. Dublin SD3246 and quality controlled. Diagnostic SNP positions were identified and abundance of these was used to estimate serovar proportions in mixed pools.

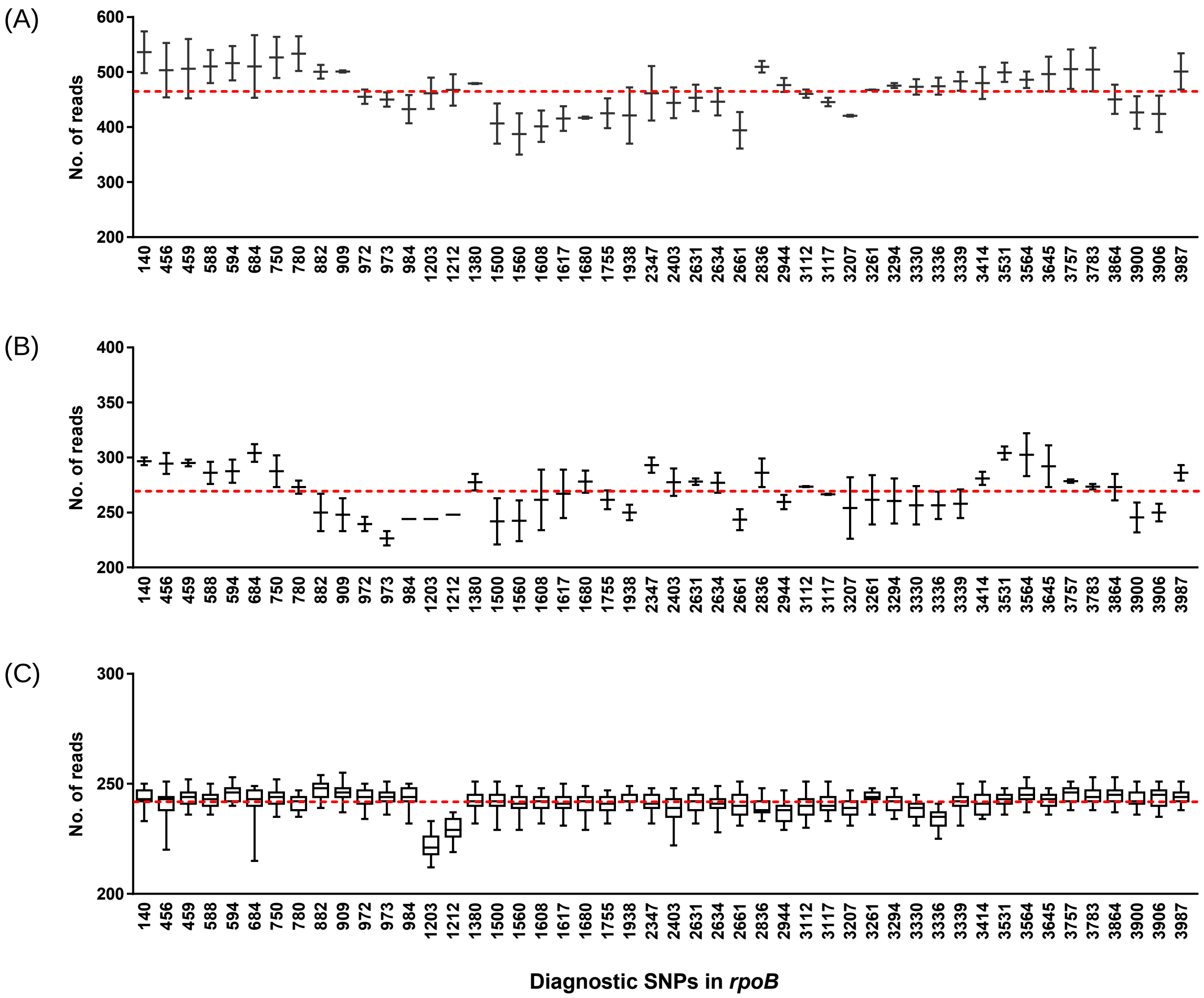

FIG S3 Read depth across *rpoB***.** The number of reads (mean, range) obtained at the chromosomal position of each diagnostic SNP in *rpoB* for all the 11 serovars used here was determined from the sequencing results. The average read depth obtained during the sequencing runs for (A) *in vitro* pools 1 and 2 (465, 350-574), (B) *in vitro* pools 3 and 4 (269, 220-322), and (C) the samples from the *in vivo* study (241, 222-247) are shown above. The dotted line represents the mean read depth across all the diagnostic SNPs.

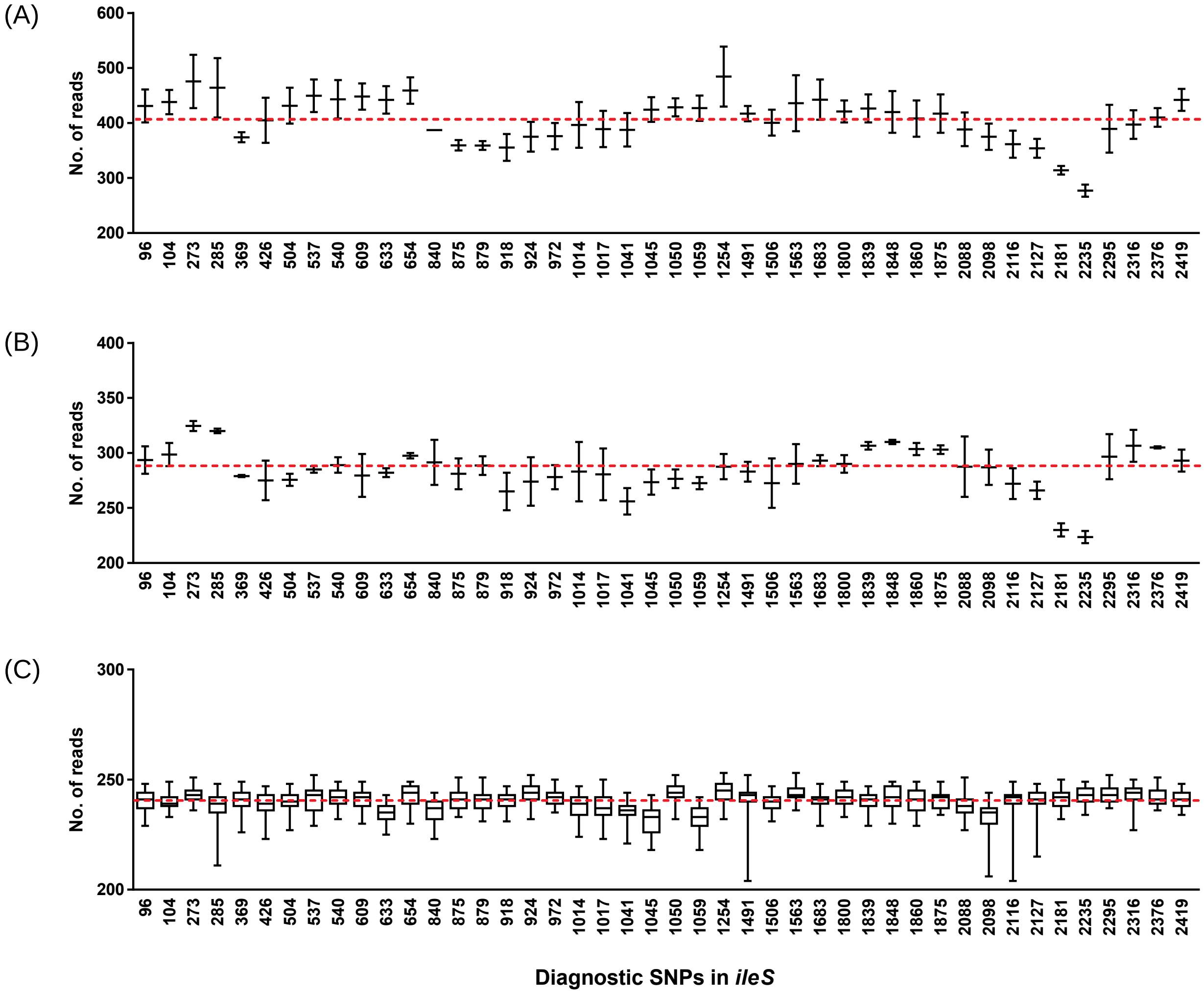

FIG S4 Read depth across *ileS*. The number of reads (mean, range) obtained at the chromosomal position of each diagnostic SNP in *ileS* for all the 11 serovars used here was determined from the sequencing results. The average read depth obtained during the sequencing runs for (A) *in vitro* pools 1 and 2 (406, 266-539), (B) *in vitro* pools 3 and 4 (288, 224-329), and (C) the samples from the *in vivo* study (240, 231-244) are shown above. The dotted line represents the mean read depth across all the diagnostic SNPs.

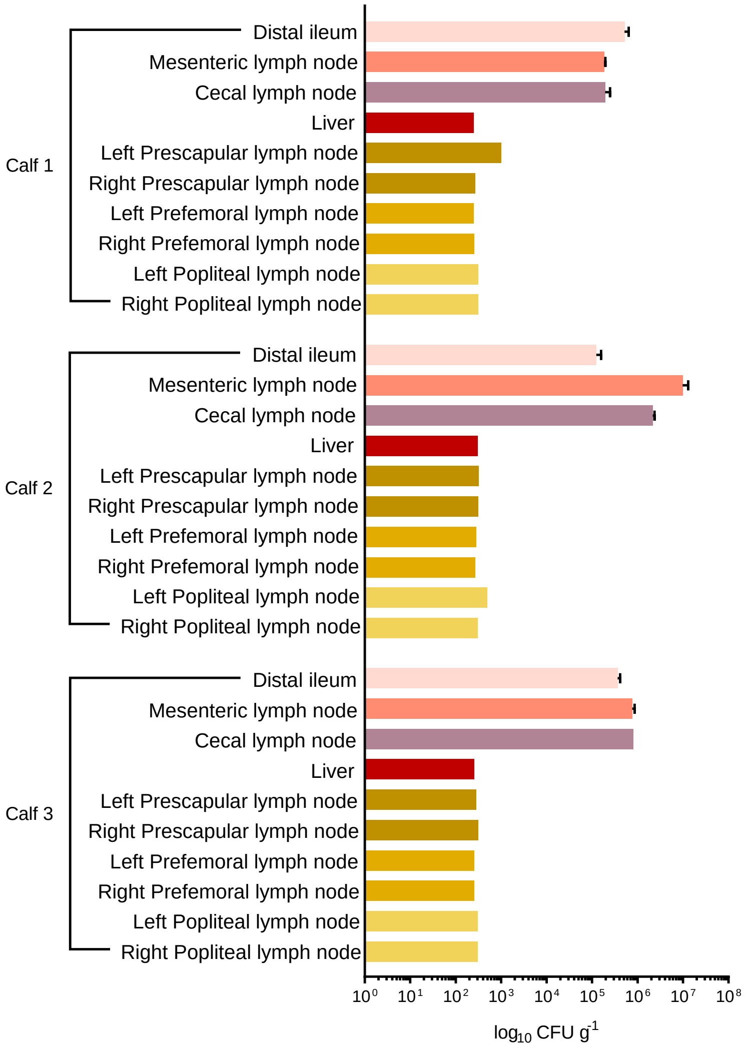

FIG S5 Bacterial load in tissues of infected calves. The distal ileum, MLNs and CLNs of all three calves orally challenged with the mixed-serovar inoculum contained approximately 10^6 CFU of *Salmonella* per gram. By contrast, the liver and PLNs only yielded approximately 300 CFU of *Salmonella* per gram.
